# Progress of the “Molecular Informatics” Section in 2022

**DOI:** 10.3390/ijms24119442

**Published:** 2023-05-29

**Authors:** Antonio Rescifina

**Affiliations:** Department of Drug and Health Sciences, University of Catania, Viale Andrea Doria 6, 95125 Catania, Italy; arescifina@unict.it

## 1. State of the Art

This is the first Editorial of the “Molecular Informatics” Section (MIS) of the *International Journal of Molecular Sciences* (*IJMS*), which was created towards the end of 2018 (the first article was submitted on 27 September 2018) and has experienced significant growth from 2018 to now. This section focuses on applying computational methods and information technology to understand molecular systems and their interactions.

In 2019, the MIS published 132 articles; by 2022, the number of published articles had more than doubled to 291. One of the key drivers of this growth has been the recent growth and development of new computational tools and methods for analyzing large datasets. For example, machine learning algorithms have been increasingly used in molecular informatics to predict the properties of molecules and understand their interactions with other molecules; molecular docking simulations, one of the key approaches of virtual screening, have been integrated with machine learning techniques to accelerate and enhance the screening process. Another notable achievement in this progress is the development of deep learning methods used to predict the activity of molecules in drug discovery. Deep learning has enabled the identification of more promising drug candidates with a higher probability of achieving clinical success by predicting the bioactivity of molecules at an unprecedented scale. These tools have allowed researchers to analyze vast amounts of data and identify patterns that would be difficult to detect through traditional experimental methods. Moreover, with the development of cloud computing platforms and the availability of powerful supercomputers, researchers can now perform complex simulations and analyses that were once impossible. Overall, the growth of MIS highlights the increasing importance of computational methods in molecular sciences. As these methods continue to advance, they are likely to play an increasingly critical role in understanding biological systems and developing new therapeutics and treatments.

From 2018, the journal impact factor (JIF) of *IJMS* grew from 4183 to 6208, with an increase of just over 48%, placing the journal in the first quartile of the Biochemistry & Molecular Biology category and the second quartile of the Multidisciplinary Chemistry category. As befits a high-level journal, this growth has rightly translated into a better and more restricted selection of the articles published in terms of quality and novelty.

The Editorial Board (EB) plays a critical role in ensuring the quality and transparency of the publication process for academic articles. The board oversees all stages of the publication process, from initial submission to final publication, and ensures that the highest standards are met.

One of the primary roles of the EB is to ensure that all submissions undergo a rigorous pre-check process to ensure they meet the journal’s submission criteria. This filter was mainly operated by the pre-check decisions of the Academic Editors (AEs), who selected articles in terms of quality and relevance to the aims and scope of the journal and, in particular, of the MIS. Moreover, the work of the AEs includes checking that the submission is original, has not been published elsewhere, and meets the required formatting and style guidelines. The guidelines of *IJMS* also encourage AEs to conduct an initial review of the submission to determine its potential suitability for publication. Once a submission has passed the pre-check process, the EB, always supported by the AEs, is responsible for selecting appropriate reviewers to assess the submission’s scientific quality and relevance. The board may consult with external experts to identify suitable reviewers and consider the author’s suggestions for potential reviewers. The EB is essential in ensuring the review process is transparent and fair. Reviewers are typically blinded to the author’s identity and the identity of other reviewers. The board is also responsible for ensuring reviewers provide constructive, respectful, and helpful feedback to the author.

To collect high-quality research articles in all fields of molecular informatics applied to the understanding of the intricate world of biology and molecular research, the Topical Collection, “Feature Papers in Molecular Informatics”, was created, and, looking only at 2022, 25 papers were published [1,2,3,4,5,6,7,8,9,10,11,12,13,14,15,16,17,18,19,20,21,22,23,24,25]. The most recent developments in computer science applied to molecular modeling have been discussed as hot topics in the two collections entitled “State-of-the-Art Molecular Informatics in Italy”, which was closed at the end of 2022 [26,27,28,29,30,31,32,33], and “Latest Review Papers in Molecular Informatics 2023”, which was closed at the end of February 2023 [34,35]. In 2022, the number of closed SIs was 22, and the top SI based on the number of published articles and visualizations was the one dedicated to “Deep Learning and Machine Learning in Bioinformatics” [36,37,38,39,40,41,42,43,44,45,46,47,48,49,50,51,52,53,54,55,56]. We also saw many publications on COVID-19 and SARS-CoV-2, with 49 papers published on this topic between 2020 and 2023 [2,10,12,57,58,59,60,61,62,63,64,65,66,67,68,69,70,71,72,73,74,75,76,77,78,79,80,81,82,83,84,85,86,87,88,89,90,91,92,93,94,95,96,97,98,99,100,101,102].

Finally, it is noteworthy to mention the five papers that have received more than one hundred citations since the birth of the MIS: (i) “Molecular Docking: Shifting Paradigms in Drug Discovery” (481 citations; review) [103], (ii) “A Structure-Based Drug Discovery Paradigm” (203 citations; review) [104], (iii) “Key Topics in Molecular Docking for Drug Design” (126 citations; review) [105], (iv) “mACPpred: A Support Vector Machine-Based Meta-Predictor for Identification of Anticancer Peptides” (115 citations; article) [106], and (v) “Investigation of Some Antiviral N-Heterocycles as COVID-19 Drug: Molecular Docking and DFT Calculations” (113 citations; article) [65].

## 2. Future Prospects

The field of molecular informatics is expected to see significant progress in 2023. With the increasing availability of molecular data, advances in machine learning and artificial intelligence tools are expected to enable more accurate predictions of molecular behavior and improved drug discovery processes. Additionally, the development of blockchain technology is expected to provide a secure, decentralized system for managing and sharing molecular data, facilitating collaborations, and accelerating research. Integrating molecular informatics with other fields, such as genomics and proteomics, is also expected to lead to innovative approaches in healthcare, personalized medicine, and drug development. It is an exciting time for the field of molecular informatics, and we can expect to see many new breakthroughs in 2023.

Despite this progress, there is still much to be achieved regarding the MIS; personally, and with the help of the entire Editorial Team (ET), in 2023, I will strive to improve the scientific quality, performance, number of publications, and visibility of the MIS as well as the JIF of *IJMS* by pursuing the development of the following points:

1. Encourage high-quality submissions: Ensure the journal clearly focuses on molecular informatics and that the published research meets high standards of originality, scientific rigor, and relevance to the field. This could be achieved by issuing a call for papers to solicit high-quality submissions and inviting leading researchers to submit manuscripts to the journal.
2. Increase the quality of published papers: The journal must implement a more rigorous peer-review process that includes multiple rounds of review by subject matter experts to improve the quality of published papers. Additionally, the journal could provide more detailed feedback to authors on areas for improvement to help them enhance the quality of their work. Another possibility may be using a double-blind review process to ensure further fairness.
3. Increase collaboration and interdisciplinary research: Encourage collaboration between molecular scientists, computer scientists, mathematicians, and bioinformaticians. This will promote interdisciplinary research to advance the field and increase the journal’s impact. Additionally, establish collaborative partnerships with relevant conferences, research organizations, and societies to increase visibility and reach within the research community.
4. Provide strong editorial guidance: The journal should have a solid ET to guide and mentor authors and reviewers. This involves identifying areas where the journal should focus, providing clear review criteria, and maintaining high publishing standards.
5. Encourage open access at low costs: Open-access publishing is the future of scientific publishing, providing greater visibility and ensuring that research reaches a broader audience. Authors could be encouraged to submit to open-access journals through waiver policies, sponsorships, or partnerships with funding agencies.
6. Prioritize quality over quantity: While increasing the number of publications is essential, it should not come at the expense of quality. Prioritize quality over quantity to maintain the journal’s reputation and impact.
7. Increase the number of publications: The journal could consider publishing more Special Issues or thematic series on topics related to the MIS to increase the number of publications. This could attract a broader range of authors and readers and encourage more submissions.
8. Increase visibility: The AE could promote the MIS through targeted marketing and social media campaigns to increase the journal’s visibility. Additionally, the journal could consider partnering with relevant scientific societies or organizations to promote the journal and its contents.
9. Increase impact factor: The editor could aim to attract high-quality research that has the potential to be cited by other researchers in the field.
10. Foster a strong community: The journal could host regular webinars, virtual conferences, or networking events focused on topics related to molecular informatics to build a strong community of authors and readers. This could help foster collaboration, knowledge-sharing, and innovation in the field.

In conclusion, great strides have been made in the progress of the MIS, which are crucial to improving *IJMS*. This progress of *IJMS* is mainly due to the proper functioning of the entire ET responsible for ensuring transparency and keeping all parties informed of the status of each submission. This includes regularly updating the author on the submission’s progress, encouraging reviewers to provide their feedback and decision timely, and making the publication process straightforward and transparent; this is what we will focus on, starting now, in the future.

With the growing focus on using artificial intelligence and machine learning in molecular computing research, further advances should automatically occur in the coming years.

## References

[1] Ali M.M., Ashraf S., Nure-e-Alam M., Qureshi U., Khan K.M., Ul-Haq Z. (2022). Identification of Selective BRD9 Inhibitor via Integrated Computational Approach. Int. J. Mol. Sci..

[2] Aliper E.T., Krylov N.A., Nolde D.E., Polyansky A.A., Efremov R.G. (2022). A Uniquely Stable Trimeric Model of SARS-CoV-2 Spike Transmembrane Domain. Int. J. Mol. Sci..

[3] Anies S., Jallu V., Diharce J., Narwani T.J., de Brevern A.G. (2022). Analysis of Integrin &alpha;IIb Subunit Dynamics Reveals Long-Range Effects of Missense Mutations on Calf Domains. Int. J. Mol. Sci..

[4] Bazzicalupi C., Bonardi A., Biver T., Ferraroni M., Papi F., Savastano M., Lombardi P., Gratteri P. (2022). Probing the Efficiency of 13-Pyridylalkyl Berberine Derivatives to Human Telomeric G-Quadruplexes Binding: Spectroscopic, Solid State and In Silico Analysis. Int. J. Mol. Sci..

[5] Chen J., Liu Z., Chang J. (2022). Genetic Mechanism Study of Auditory Phoenix Spheres and Transcription Factors Prediction for Direct Reprogramming by Bioinformatics. Int. J. Mol. Sci..

[6] Cheng Y., Luo J., Li H., Wei F., Zhang Y., Jiang H., Peng X. (2022). Identification of the WRKY Gene Family and Characterization of Stress-Responsive Genes in Taraxacum kok-saghyz Rodin. Int. J. Mol. Sci..

[7] Csávás M., Kalmár L., Szőke P., Farkas L.B., Bécsi B., Kónya Z., Kerékgyártó J., Borbás A., Erdődi F., Kövér K.E. (2022). A Fucosylated Lactose-Presenting Tetravalent Glycocluster Acting as a Mutual Ligand of Pseudomonas aeruginosa Lectins A (PA-IL) and B (PA-IIL)&mdash;Synthesis and Interaction Studies. Int. J. Mol. Sci..

[8] Curreli F., Kwon Y.D., Nicolau I., Burgos G., Altieri A., Kurkin A.V., Verardi R., Kwong P.D., Debnath A.K. (2022). Antiviral Activity and Crystal Structures of HIV-1 gp120 Antagonists. Int. J. Mol. Sci..

[9] de Brevern A.G. (2022). A Perspective on the (Rise and Fall of) Protein β-Turns. Int. J. Mol. Sci..

[10] Defant A., Dosi F., Innocenti N., Mancini I. (2022). Synthesis of Nucleoside-like Molecules from a Pyrolysis Product of Cellulose and Their Computational Prediction as Potential SARS-CoV-2 RNA-Dependent RNA Polymerase Inhibitors. Int. J. Mol. Sci..

[11] Enrich-Bengoa J., Manich G., Dégano I.R., Perálvarez-Marín A. (2022). Deciphering the Genetic Crosstalk between Microglia and Oligodendrocyte Precursor Cells during Demyelination and Remyelination Using Transcriptomic Data. Int. J. Mol. Sci..

[12] Floresta G., Zagni C., Gentile D., Patamia V., Rescifina A. (2022). Artificial Intelligence Technologies for COVID-19 De Novo Drug Design. Int. J. Mol. Sci..

[13] Gómez Borrego J., Torrent Burgas M. (2022). Analysis of Host&ndash;Bacteria Protein Interactions Reveals Conserved Domains and Motifs That Mediate Fundamental Infection Pathways. Int. J. Mol. Sci..

[14] Gonzalez-Bosquet J., Gabrilovich S., McDonald M.E., Smith B.J., Leslie K.K., Bender D.D., Goodheart M.J., Devor E. (2022). Integration of Genomic and Clinical Retrospective Data to Predict Endometrioid Endometrial Cancer Recurrence. Int. J. Mol. Sci..

[15] He J., Wu Y., Pu X., Li M., Guo Y. (2022). A Transfer-Learning-Based Deep Convolutional Neural Network for Predicting Leukemia-Related Phosphorylation Sites from Protein Primary Sequences. Int. J. Mol. Sci..

[16] Ielo L., Patamia V., Citarella A., Efferth T., Shahhamzehei N., Schirmeister T., Stagno C., Langer T., Rescifina A., Micale N. (2022). Novel Class of Proteasome Inhibitors: In Silico and In Vitro Evaluation of Diverse Chloro(trifluoromethyl)aziridines. Int. J. Mol. Sci..

[17] Jin C., Shi Z., Kang C., Lin K., Zhang H. (2022). TLCrys: Transfer Learning Based Method for Protein Crystallization Prediction. Int. J. Mol. Sci..

[18] Lebel M., Very T., Gloaguen E., Tardivel B., Mons M., Brenner V. (2022). Excited States Computation of Models of Phenylalanine Protein Chains: TD-DFT and Composite CC2/TD-DFT Protocols. Int. J. Mol. Sci..

[19] Miura N., Hanamatsu H., Yokota I., Akasaka-Manya K., Manya H., Endo T., Shinohara Y., Furukawa J.-I. (2022). Toolbox Accelerating Glycomics (TAG): Improving Large-Scale Serum Glycomics and Refinement to Identify SALSA-Modified and Rare Glycans. Int. J. Mol. Sci..

[20] Patamia V., Floresta G., Pistarà V., Rescifina A. (2022). Green Efficient One-Pot Synthesis and Separation of Nitrones in Water Assisted by a Self-Assembled Nanoreactor. Int. J. Mol. Sci..

[21] Pestana-Nobles R., Aranguren-Díaz Y., Machado-Sierra E., Yosa J., Galan-Freyle N.J., Sepulveda-Montaño L.X., Kuroda D.G., Pacheco-Londoño L.C. (2022). Docking and Molecular Dynamic of Microalgae Compounds as Potential Inhibitors of Beta-Lactamase. Int. J. Mol. Sci..

[22] Rissanou A., Karnis I., Krasanakis F., Chrissopoulou K., Karatasos K. (2022). The Role of Oxidation Pattern and Water Content in the Spatial Arrangement and Dynamics of Oxidized Graphene-Based Aqueous Dispersions. Int. J. Mol. Sci..

[23] Rusanov A.I., Dmitrieva O.A., Mamardashvili N.Z., Tetko I.V. (2022). More Is Not Always Better: Local Models Provide Accurate Predictions of Spectral Properties of Porphyrins. Int. J. Mol. Sci..

[24] Tziastoudi M., Cholevas C., Theoharides T.C., Stefanidis I. (2022). Meta-Analysis and Bioinformatics Detection of Susceptibility Genes in Diabetic Nephropathy. Int. J. Mol. Sci..

[25] Vázquez-Durán A., Téllez-Isaías G., Hernández-Rodríguez M., Ruvalcaba R.M., Martínez J., Nicolás-Vázquez M.I., Aceves-Hernández J.M., Méndez-Albores A. (2022). The Ability of Chlorophyll to Trap Carcinogen Aflatoxin B1: A Theoretical Approach. Int. J. Mol. Sci..

[26] Ciriaco F., Gambacorta N., Trisciuzzi D., Nicolotti O. (2022). PLATO: A Predictive Drug Discovery Web Platform for Efficient Target Fishing and Bioactivity Profiling of Small Molecules. Int. J. Mol. Sci..

[27] D’Arrigo G., Autiero I., Gianquinto E., Siragusa L., Baroni M., Cruciani G., Spyrakis F. (2022). Exploring Ligand Binding Domain Dynamics in the NRs Superfamily. Int. J. Mol. Sci..

[28] Fassi E.M.A., Garofalo M., Sgrignani J., Dei Cas M., Mori M., Roda G., Cavalli A., Grazioso G. (2022). Focused Design of Novel Cyclic Peptides Endowed with GABARAP-Inhibiting Activity. Int. J. Mol. Sci..

[29] Gervasoni S., Talarico C., Manelfi C., Pedretti A., Vistoli G., Beccari A.R. (2022). Extensive Sampling of Molecular Dynamics Simulations to Identify Reliable Protein Structures for Optimized Virtual Screening Studies: The Case of the hTRPM8 Channel. Int. J. Mol. Sci..

[30] Mammoli A., Bianconi E., Ruta L., Riccio A., Bigiotti C., Souma M., Carotti A., Rossini S., Suvieri C., Pallotta M.T. (2022). Critical Assessment of a Structure-Based Screening Campaign for IDO1 Inhibitors: Tips and Pitfalls. Int. J. Mol. Sci..

[31] Mauri A., Bertola M. (2022). Alvascience: A New Software Suite for the QSAR Workflow Applied to the Blood&ndash;Brain Barrier Permeability. Int. J. Mol. Sci..

[32] Selvestrel G., Lavado G.J., Toropova A.P., Toropov A.A., Gadaleta D., Marzo M., Baderna D., Benfenati E. (2022). Monte Carlo Models for Sub-Chronic Repeated-Dose Toxicity: Systemic and Organ-Specific Toxicity. Int. J. Mol. Sci..

[33] Patamia V., Floresta G., Zagni C., Pistarà V., Punzo F., Rescifina A. (2023). 1,2-Dibenzoylhydrazine as a Multi-Inhibitor Compound: A Morphological and Docking Study. Int. J. Mol. Sci..

[34] Patamia V., Zagni C., Brullo I., Saccullo E., Coco A., Floresta G., Rescifina A. (2023). Computer-Assisted Design of Peptide-Based Radiotracers. Int. J. Mol. Sci..

[35] Tran C., Khadkikar S., Porollo A. (2023). Survey of Protein Sequence Embedding Models. Int. J. Mol. Sci..

[36] Le N.Q.K., Do D.T., Hung T.N.K., Lam L.H.T., Huynh T.-T., Nguyen N.T.K. (2020). A Computational Framework Based on Ensemble Deep Neural Networks for Essential Genes Identification. Int. J. Mol. Sci..

[37] Al Mamun A., Tanvir R.B., Sobhan M., Mathee K., Narasimhan G., Holt G.E., Mondal A.M. (2021). Multi-Run Concrete Autoencoder to Identify Prognostic lncRNAs for 12 Cancers. Int. J. Mol. Sci..

[38] Alessandri L., Ratto M.L., Contaldo S.G., Beccuti M., Cordero F., Arigoni M., Calogero R.A. (2021). Sparsely Connected Autoencoders: A Multi-Purpose Tool for Single Cell omics Analysis. Int. J. Mol. Sci..

[39] Auliah F.N., Nilamyani A.N., Shoombuatong W., Alam M.A., Hasan M.M., Kurata H. (2021). PUP-Fuse: Prediction of Protein Pupylation Sites by Integrating Multiple Sequence Representations. Int. J. Mol. Sci..

[40] Auslander N., Gussow A.B., Koonin E.V. (2021). Incorporating Machine Learning into Established Bioinformatics Frameworks. Int. J. Mol. Sci..

[41] Banegas-Luna A.J., Peña-García J., Iftene A., Guadagni F., Ferroni P., Scarpato N., Zanzotto F.M., Bueno-Crespo A., Pérez-Sánchez H. (2021). Towards the Interpretability of Machine Learning Predictions for Medical Applications Targeting Personalised Therapies: A Cancer Case Survey. Int. J. Mol. Sci..

[42] Campos T.L., Korhonen P.K., Young N.D. (2021). Cross-Predicting Essential Genes between Two Model Eukaryotic Species Using Machine Learning. Int. J. Mol. Sci..

[43] Charoenkwan P., Nantasenamat C., Hasan M.M., Moni M.A., Lio’ P., Shoombuatong W. (2021). iBitter-Fuse: A Novel Sequence-Based Bitter Peptide Predictor by Fusing Multi-View Features. Int. J. Mol. Sci..

[44] Defresne M., Barbe S., Schiex T. (2021). Protein Design with Deep Learning. Int. J. Mol. Sci..

[45] Del Giudice M., Peirone S., Perrone S., Priante F., Varese F., Tirtei E., Fagioli F., Cereda M. (2021). Artificial Intelligence in Bulk and Single-Cell RNA-Sequencing Data to Foster Precision Oncology. Int. J. Mol. Sci..

[46] Jabeen A., de March C.A., Matsunami H., Ranganathan S. (2021). Machine Learning Assisted Approach for Finding Novel High Activity Agonists of Human Ectopic Olfactory Receptors. Int. J. Mol. Sci..

[47] Lee B., Shin M.K., Hwang I.-W., Jung J., Shim Y.J., Kim G.W., Kim S.T., Jang W., Sung J.-S. (2021). A Deep Learning Approach with Data Augmentation to Predict Novel Spider Neurotoxic Peptides. Int. J. Mol. Sci..

[48] Madani M., Lin K., Tarakanova A. (2021). DSResSol: A Sequence-Based Solubility Predictor Created with Dilated Squeeze Excitation Residual Networks. Int. J. Mol. Sci..

[49] Nilamyani A.N., Auliah F.N., Moni M.A., Shoombuatong W., Hasan M.M., Kurata H. (2021). PredNTS: Improved and Robust Prediction of Nitrotyrosine Sites by Integrating Multiple Sequence Features. Int. J. Mol. Sci..

[50] Nosi V., Luca A., Milan M., Arigoni M., Benvenuti S., Cacchiarelli D., Cesana M., Riccardo S., Di Filippo L., Cordero F. (2021). MET Exon 14 Skipping: A Case Study for the Detection of Genetic Variants in Cancer Driver Genes by Deep Learning. Int. J. Mol. Sci..

[51] Persson Hodén K., Hu X., Martinez G., Dixelius C. (2021). smartPARE: An R Package for Efficient Identification of True mRNA Cleavage Sites. Int. J. Mol. Sci..

[52] Rodin A.S., Gogoshin G., Hilliard S., Wang L., Egelston C., Rockne R.C., Chao J., Lee P.P. (2021). Dissecting Response to Cancer Immunotherapy by Applying Bayesian Network Analysis to Flow Cytometry Data. Int. J. Mol. Sci..

[53] Hazra D., Kim M.-R., Byun Y.-C. (2022). Generative Adversarial Networks for Creating Synthetic Nucleic Acid Sequences of Cat Genome. Int. J. Mol. Sci..

[54] Pouryahya M., Oh J.H., Mathews J.C., Belkhatir Z., Moosmüller C., Deasy J.O., Tannenbaum A.R. (2022). Pan-Cancer Prediction of Cell-Line Drug Sensitivity Using Network-Based Methods. Int. J. Mol. Sci..

[55] Roethel A., Biliński P., Ishikawa T. (2022). BioS2Net: Holistic Structural and Sequential Analysis of Biomolecules Using a Deep Neural Network. Int. J. Mol. Sci..

[56] Zulfiqar H., Huang Q.-L., Lv H., Sun Z.-J., Dao F.-Y., Lin H. (2022). Deep-4mCGP: A Deep Learning Approach to Predict 4mC Sites in Geobacter pickeringii by Using Correlation-Based Feature Selection Technique. Int. J. Mol. Sci..

[57] Bergonzo C., Szakal A.L. (2020). Using All-Atom Potentials to Refine RNA Structure Predictions of SARS-CoV-2 Stem Loops. Int. J. Mol. Sci..

[58] Cannalire R., Stefanelli I., Cerchia C., Beccari A.R., Pelliccia S., Summa V. (2020). SARS-CoV-2 Entry Inhibitors: Small Molecules and Peptides Targeting Virus or Host Cells. Int. J. Mol. Sci..

[59] Fischer A., Sellner M., Neranjan S., Smieško M., Lill M.A. (2020). Potential Inhibitors for Novel Coronavirus Protease Identified by Virtual Screening of 606 Million Compounds. Int. J. Mol. Sci..

[60] Gand M., Vanneste K., Thomas I., Van Gucht S., Capron A., Herman P., Roosens N.H.C., De Keersmaecker S.C.J. (2020). Use of Whole Genome Sequencing Data for a First in Silico Specificity Evaluation of the RT-qPCR Assays Used for SARS-CoV-2 Detection. Int. J. Mol. Sci..

[61] Gentile D., Fuochi V., Rescifina A., Furneri P.M. (2020). New Anti SARS-Cov-2 Targets for Quinoline Derivatives Chloroquine and Hydroxychloroquine. Int. J. Mol. Sci..

[62] Gervasoni S., Vistoli G., Talarico C., Manelfi C., Beccari A.R., Studer G., Tauriello G., Waterhouse A.M., Schwede T., Pedretti A. (2020). A Comprehensive Mapping of the Druggable Cavities within the SARS-CoV-2 Therapeutically Relevant Proteins by Combining Pocket and Docking Searches as Implemented in Pockets 2.0. Int. J. Mol. Sci..

[63] Gimeno A., Mestres-Truyol J., Ojeda-Montes M.J., Macip G., Saldivar-Espinoza B., Cereto-Massagué A., Pujadas G., Garcia-Vallvé S. (2020). Prediction of Novel Inhibitors of the Main Protease (M-pro) of SARS-CoV-2 through Consensus Docking and Drug Reposition. Int. J. Mol. Sci..

[64] Grottesi A., Bešker N., Emerson A., Manelfi C., Beccari A.R., Frigerio F., Lindahl E., Cerchia C., Talarico C. (2020). Computational Studies of SARS-CoV-2 3CLpro: Insights from MD Simulations. Int. J. Mol. Sci..

[65] Hagar M., Ahmed H.A., Aljohani G., Alhaddad O.A. (2020). Investigation of Some Antiviral N-Heterocycles as COVID 19 Drug: Molecular Docking and DFT Calculations. Int. J. Mol. Sci..

[66] Lee A.C., Chakladar J., Li W.T., Chen C., Chang E.Y., Wang-Rodriguez J., Ongkeko W.M. (2020). Tobacco, but Not Nicotine and Flavor-Less Electronic Cigarettes, Induces ACE2 and Immune Dysregulation. Int. J. Mol. Sci..

[67] Srivastava R., Daulatabad S.V., Srivastava M., Janga S.C. (2020). Role of SARS-CoV-2 in Altering the RNA-Binding Protein and miRNA-Directed Post-Transcriptional Regulatory Networks in Humans. Int. J. Mol. Sci..

[68] Battaglia R., Alonzo R., Pennisi C., Caponnetto A., Ferrara C., Stella M., Barbagallo C., Barbagallo D., Ragusa M., Purrello M. (2021). MicroRNA-Mediated Regulation of the Virus Cycle and Pathogenesis in the SARS-CoV-2 Disease. Int. J. Mol. Sci..

[69] Borocci S., Cerchia C., Grottesi A., Sanna N., Prandi I.G., Abid N., Beccari A.R., Chillemi G., Talarico C. (2021). Altered Local Interactions and Long-Range Communications in UK Variant (B.1.1.7) Spike Glycoprotein. Int. J. Mol. Sci..

[70] Brullo C., Villa C., Tasso B., Russo E., Spallarossa A. (2021). Btk Inhibitors: A Medicinal Chemistry and Drug Delivery Perspective. Int. J. Mol. Sci..

[71] Cho T., Han H.-S., Jeong J., Park E.-M., Shim K.-S. (2021). A Novel Computational Approach for the Discovery of Drug Delivery System Candidates for COVID-19. Int. J. Mol. Sci..

[72] Citarella A., Gentile D., Rescifina A., Piperno A., Mognetti B., Gribaudo G., Sciortino M.T., Holzer W., Pace V., Micale N. (2021). Pseudo-Dipeptide Bearing α,α-Difluoromethyl Ketone Moiety as Electrophilic Warhead with Activity against Coronaviruses. Int. J. Mol. Sci..

[73] Formanowicz D., Gutowska K., Szawulak B., Formanowicz P. (2021). The Crosstalk between SARS-CoV-2 Infection and the RAA System in Essential Hypertension—Analyses Using Systems Approach. Int. J. Mol. Sci..

[74] Galanis K.A., Nastou K.C., Papandreou N.C., Petichakis G.N., Pigis D.G., Iconomidou V.A. (2021). Linear B-Cell Epitope Prediction for In Silico Vaccine Design: A Performance Review of Methods Available via Command-Line Interface. Int. J. Mol. Sci..

[75] Mekni N., Coronnello C., Langer T., Rosa M.D., Perricone U. (2021). Support Vector Machine as a Supervised Learning for the Prioritization of Novel Potential SARS-CoV-2 Main Protease Inhibitors. Int. J. Mol. Sci..

[76] Morena F., Argentati C., Tortorella I., Emiliani C., Martino S. (2021). De novo ssRNA Aptamers against the SARS-CoV-2 Main Protease: In Silico Design and Molecular Dynamics Simulation. Int. J. Mol. Sci..

[77] Peralta-Garcia A., Torrens-Fontanals M., Stepniewski T.M., Grau-Expósito J., Perea D., Ayinampudi V., Waldhoer M., Zimmermann M., Buzón M.J., Genescà M. (2021). Entrectinib&mdash;A SARS-CoV-2 Inhibitor in Human Lung Tissue (HLT) Cells. Int. J. Mol. Sci..

[78] Silva R.C., Freitas H.F., Campos J.M., Kimani N.M., Silva C.H.T.P., Borges R.S., Pita S.S.R., Santos C.B.R. (2021). Natural Products-Based Drug Design against SARS-CoV-2 Mpro 3CLpro. Int. J. Mol. Sci..

[79] Sojka D., Šnebergerová P., Robbertse L. (2021). Protease Inhibition—An Established Strategy to Combat Infectious Diseases. Int. J. Mol. Sci..

[80] Stasiulewicz A., Maksymiuk A.W., Nguyen M.L., Bełza B., Sulkowska J.I. (2021). SARS-CoV-2 Papain-Like Protease Potential Inhibitors—In Silico Quantitative Assessment. Int. J. Mol. Sci..

[81] Tagliamonte M.S., Abid N., Borocci S., Sangiovanni E., Ostrov D.A., Kosakovsky Pond S.L., Salemi M., Chillemi G., Mavian C. (2021). Multiple Recombination Events and Strong Purifying Selection at the Origin of SARS-CoV-2 Spike Glycoprotein Increased Correlated Dynamic Movements. Int. J. Mol. Sci..

[82] Zhang R., Liu Y., Zhang X., Xiao K., Hou Y., Liu H., Sun X. (2021). Detecting and Profiling Endogenous RNA G-Quadruplexes in the Human Transcriptome. Int. J. Mol. Sci..

[83] Chaves O.A., Rodrigues-Santos C.E., Echevarria Á., Sacramento C.Q., Fintelman-Rodrigues N., Temerozo J.R., Castro-Faria-Neto H.C., Souza T.M.L.e. (2022). Fluorine Atoms on C6H5-Corrole Affect the Interaction with Mpro and PLpro Proteases of SARS-CoV-2: Molecular Docking and 2D-QSAR Approaches. Int. J. Mol. Sci..

[84] D’Amato M., Iadarola P., Viglio S. (2022). Proteomic Analysis of Human Sputum for the Diagnosis of Lung Disorders: Where Are We Today?. Int. J. Mol. Sci..

[85] Dassanayake M.K., Khoo T.-J., Chong C.H., Di Martino P. (2022). Molecular Docking and In-Silico Analysis of Natural Biomolecules against Dengue, Ebola, Zika, SARS-CoV-2 Variants of Concern and Monkeypox Virus. Int. J. Mol. Sci..

[86] Dukeshire M., Schaeper D., Venkatesan P., Manzourolajdad A. (2022). Variant-Specific Analysis Reveals a Novel Long-Range RNA-RNA Interaction in SARS-CoV-2 Orf1a. Int. J. Mol. Sci..

[87] Duma Z., Ramsuran V., Chuturgoon A.A., Edward V.A., Naidoo P., Mkhize-Kwitshana Z.L. (2022). Evaluation of Various Alternative Economical and High Throughput SARS-CoV-2 Testing Methods within Resource-Limited Settings. Int. J. Mol. Sci..

[88] Elkaeed E.B., Eissa I.H., Elkady H., Abdelalim A., Alqaisi A.M., Alsfouk A.A., Elwan A., Metwaly A.M. (2022). A Multistage In Silico Study of Natural Potential Inhibitors Targeting SARS-CoV-2 Main Protease. Int. J. Mol. Sci..

[89] Elkaeed E.B., Youssef F.S., Eissa I.H., Elkady H., Alsfouk A.A., Ashour M.L., El Hassab M.A., Abou-Seri S.M., Metwaly A.M. (2022). Multi-Step In Silico Discovery of Natural Drugs against COVID-19 Targeting Main Protease. Int. J. Mol. Sci..

[90] Flora J., Khan W., Jin J., Jin D., Hussain A., Dajani K., Khan B. (2022). Usefulness of Vaccine Adverse Event Reporting System for Machine-Learning Based Vaccine Research: A Case Study for COVID-19 Vaccines. Int. J. Mol. Sci..

[91] Gentile D., Coco A., Patamia V., Zagni C., Floresta G., Rescifina A. (2022). Targeting the SARS-CoV-2 HR1 with Small Molecules as Inhibitors of the Fusion Process. Int. J. Mol. Sci..

[92] Gregori J., Colomer-Castell S., Campos C., Ibañez-Lligoña M., Garcia-Cehic D., Rando-Segura A., Adombie C.M., Pintó R., Guix S., Bosch A. (2022). Quasispecies Fitness Partition to Characterize the Molecular Status of a Viral Population. Negative Effect of Early Ribavirin Discontinuation in a Chronically Infected HEV Patient. Int. J. Mol. Sci..

[93] Liao Y.-C., Chen F.-J., Chuang M.-C., Wu H.-C., Ji W.-C., Yu G.-Y., Huang T.-S. (2022). High-Integrity Sequencing of Spike Gene for SARS-CoV-2 Variant Determination. Int. J. Mol. Sci..

[94] Nalewaj M., Szabat M. (2022). Examples of Structural Motifs in Viral Genomes and Approaches for RNA Structure Characterization. Int. J. Mol. Sci..

[95] Preet G., Oluwabusola E.T., Milne B.F., Ebel R., Jaspars M. (2022). Computational Repurposing of Mitoxantrone-Related Structures against Monkeypox Virus: A Molecular Docking and 3D Pharmacophore Study. Int. J. Mol. Sci..

[96] Qiu Y., Liu Q., Tu G., Yao X.-J. (2022). Discovery of the Cryptic Sites of SARS-CoV-2 Papain-like Protease and Analysis of Its Druggability. Int. J. Mol. Sci..

[97] Ramos R.S., Borges R.S., de Souza J.S.N., Araujo I.F., Chaves M.H., Santos C.B.R. (2022). Identification of Potential Antiviral Inhibitors from Hydroxychloroquine and 1,2,4,5-Tetraoxanes Analogues and Investigation of the Mechanism of Action in SARS-CoV-2. Int. J. Mol. Sci..

[98] Saik O.V., Klimontov V.V. (2022). Gene Networks of Hyperglycemia, Diabetic Complications, and Human Proteins Targeted by SARS-CoV-2: What Is the Molecular Basis for Comorbidity?. Int. J. Mol. Sci..

[99] Daamen A.R., Bachali P., Grammer A.C., Lipsky P.E. (2023). Classification of COVID-19 Patients into Clinically Relevant Subsets by a Novel Machine Learning Pipeline Using Transcriptomic Features. Int. J. Mol. Sci..

[100] Laterza L., Putignani L., Settanni C.R., Petito V., Varca S., De Maio F., Macari G., Guarrasi V., Gremese E., Tolusso B. (2023). Ecology and Machine Learning-Based Classification Models of Gut Microbiota and Inflammatory Markers May Evaluate the Effects of Probiotic Supplementation in Patients Recently Recovered from COVID-19. Int. J. Mol. Sci..

[101] Menin S., Pavan M., Salmaso V., Sturlese M., Moro S. (2023). Thermal Titration Molecular Dynamics (TTMD): Not Your Usual Post-Docking Refinement. Int. J. Mol. Sci..

[102] Zeng L., Lu Y., Yan W., Yang Y. (2023). A Protein Co-Conservation Network Model Characterizes Mutation Effects on SARS-CoV-2 Spike Protein. Int. J. Mol. Sci..

[103] Pinzi L., Rastelli G. (2019). Molecular Docking: Shifting Paradigms in Drug Discovery. Int. J. Mol. Sci..

[104] Batool M., Ahmad B., Choi S. (2019). A Structure-Based Drug Discovery Paradigm. Int. J. Mol. Sci..

[105] Torres P.H.M., Sodero A.C.R., Jofily P., Silva-Jr F.P. (2019). Key Topics in Molecular Docking for Drug Design. Int. J. Mol. Sci..

[106] Boopathi V., Subramaniyam S., Malik A., Lee G., Manavalan B., Yang D.-C. (2019). mACPpred: A Support Vector Machine-Based Meta-Predictor for Identification of Anticancer Peptides. Int. J. Mol. Sci..

